# Addressing Infectious Diseases in Vulnerable Populations Under the Auspices of One Health: A Call for Action in Europe

**DOI:** 10.3390/idr18010012

**Published:** 2026-01-15

**Authors:** Botond Lakatos, Ferenc Balázs Farkas, Giacomo Guido, Annalisa Saracino, Francesco Di Gennaro

**Affiliations:** 1 Department of Hematology and Infectious Diseases, School of Infectious Diseases, Semmelweis University, 1097 Budapest, Hungary; 2 Department of Infectious Diseases, National Institute of Hematology and Infectious Diseases, 1097 Budapest, Hungary; 3 Tropical Medicine and Global Health Working Group, Semmelweis University, 1097 Budapest, Hungary; 4 Pediatric Center, Semmelweis University, 1083 Budapest, Hungary; 5 Institute of Medical Microbiology, Semmelweis University, 1089 Budapest, Hungary; 6 Clinic of Infectious Diseases, Department of Precision and Regenerative Medicine and Ionian Area (DiMePRe-J), University of Bari “Aldo Moro”, 70124 Bari, Italy

## 1. Background

While infectious diseases represent a daunting challenge to public health worldwide, their impact is disproportionately felt among the most vulnerable and marginalized segments of society. Over the past decade, disadvantaged socioeconomic phenomena, armed conflicts, the COVID-19 pandemic and surging natural disasters have pushed millions of people globally into extreme poverty according to the World Bank [1,2]. In the European Union, about 20–22% of the population (around 95 million people) were at risk of poverty or social exclusion in the past few years as defined by Eurostat [3].

**Figure 1:**
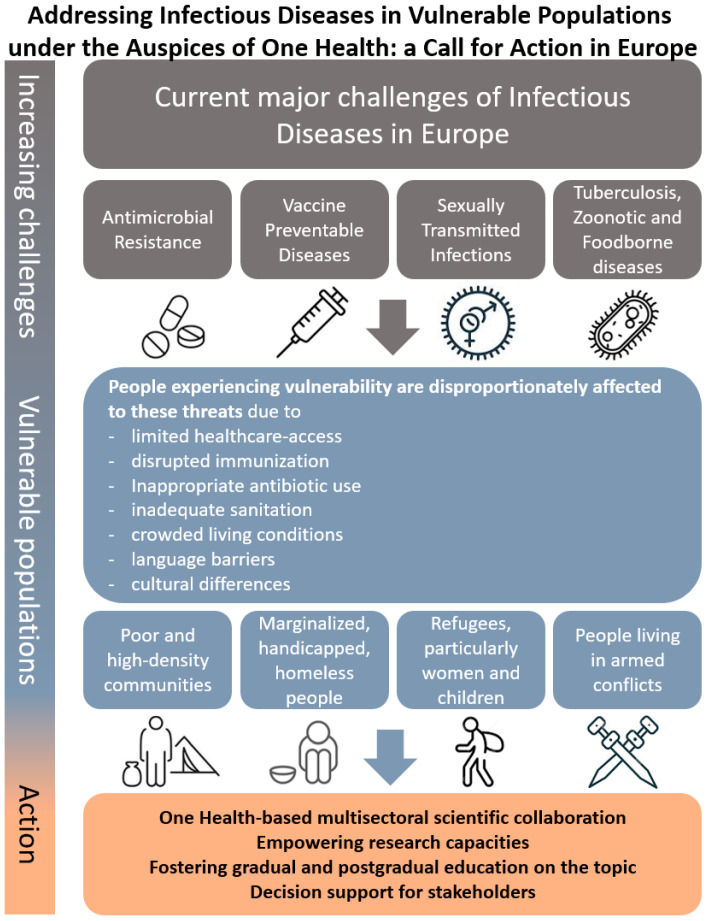
Increasing threats of infectious diseases affect disproportionately vulnerable populations in Europe, therefore joint multisectoral actions are warranted.

In parallel, Europe has experienced infectious disease-related challenges: epidemics in vaccine-preventable diseases like pertussis, measles, and hepatitis A, and in some sexually transmitted infections (STIs), particularly syphilis, HIV and gonorrhea. Additionally, Antimicrobial Resistance (AMR) is a notable problem; tuberculosis, some zoonotic and vector-borne diseases remain a serious cause of concern [4,5,6,7,8,9,10,11,12]. Most vulnerable to these epidemics are socioeconomically compromised individuals facing existential uncertainties, limited access to healthcare, disrupted vaccination schedules, inappropriate antibiotic use, sometimes with inadequate sanitation and crowded living conditions (Figure 1). Given the multiple layers of the challenges, a comprehensive, multisectoral approach might only be successfully applicable at both regional and global levels. The One Health approach proved to be an effective way to join efforts and provide a platform for collaboration for human, animal and environmental professionals. Recognizing this need, the European Commission hosted a Cross-agency One Health Task Force in May 2025 to foster coordination and implementation of the One Health approach in the European Union [13]. This commentary aims to stress that in spite of advancements, infectious diseases are a high risk for the most vulnerable people, whereas a strengthened and intersectoral One Health paradigm may provide benefit.

## 2. Populations Vulnerable to Infectious Diseases

Defining vulnerability and vulnerable populations is challenging seeing that they constitute an extremely heterogeneous group of exposed people. Notably, there is no straightforward definition for the phenomenon of vulnerable populations of infectious diseases [14]. Meanwhile, the World Health Organization provides a definition in the context of people vulnerable to hazards [15,16]. With the adaptation of that, a definition for vulnerability in the context of infectious diseases is the following: *“The conditions determined by physical, mental, social, economic, and environmental factors which increase the susceptibility of an individual and a community to the impacts of infectious diseases and epidemics or pandemics.”* It needs to be emphasized that the contextual and social determinants are cardinal, differentiating the susceptibility to infections of a “classical” immunocompromised patient, where the susceptibility to infections is almost solely based on the patient’s immunological pattern largely unrelated to the socioeconomic environment. Based on the Canadian National Collaborating Centre for Determinants of Health, vulnerability occurs when *“people are exposed to multiple layers of marginalization, including barriers to social, economic, political and environmental resources that overlap to increase the risk of poor health”* [17].

*Poor and high-density* communities will continue to bear the brunt of outbreaks, leading to broader health crises, if they do not receive significant investment in public health infrastructure and education [18]. *People experiencing homelessness, refugees and immigrants* are often linked to compromised health due to exposure to the elements, food insecurity, and insufficient access to healthcare, social segregation, and potentially underlying mental health issues [19,20]. Women are at an added risk of sexual violence and exploitation, particularly sex workers, which can further exacerbate their health challenges. In the case of *marginalized communities*, culturally competent healthcare solutions must be prioritized to engage these populations effectively. *People with mental or physical disabilities* face unique barriers that heighten their risk for infectious diseases. In all these scenarios, further barriers to the appropriate medical assistance are imposed due to their status as marginalized individuals of society, stigmatization or self-stigmatization. Lastly, but more importantly, *people living in zones of armed conflict* endure a catastrophic convergence of risk factors for infectious diseases. The breakdown of healthcare systems together with food shortages and the destruction of infrastructure create fertile ground for outbreaks. Current armed conflicts in Ukraine have resulted in a total humanitarian catastrophe, with unforeseen long-term consequences, which needs to be addressed with urgency, providing immediate relief [21,22,23]. Notably, rising rates of multiresistant bacteria, particularly high numbers of New Delhi metallo-β-lactamase producer Gram-negative bacteria have been noted in war-wounded soldiers and civil population in Ukraine and in other European countries taking in Ukrainian war refugees [21,24,25].

## 3. Potential to Move Ahead

When it comes to these issues, an obvious escape route does not exist. There is no unified advocacy as this is a heterogeneous group of marginalized people and a neglected topic. There is a limited number of high-level authentic scientific publications on the topics of prevention, detection and management of infectious diseases in vulnerable populations. Apparently, a robust research, grounded and scientifically justified multi-faceted approach to address infectious diseases in vulnerable populations is warranted. Based on the findings, reconsidering the immediate interventions and long-term strategies will support identifying and handling the roots of the complex scenarios. Addressing these inequities is not only a moral imperative but also essential for the overall health of our communities, particularly in the context of One Health. Therefore, we propose that a European network of dedicated interdisciplinary human, animal and environmental health professionals should focus on the infections of the vulnerable populations, where the One Health approach stays on the frontline.

Furthermore, medical education and training curricula for physicians, nurses, community workers, public health and other health professionals should evolve to include both technical knowledge and cultural sensitivity regarding vulnerable populations [25]. Future generations of physicians must be equipped not only with clinical skills but also with an understanding of the complex social and structural determinants of infectious diseases. This will advance their capacity to serve disadvantaged communities with competence, empathy, and lasting impact [26]. Notably, transmission of reliable education is even more substantial knowing that ChatGPT (Version 4, Open AI, San Francisco, CA, USA), the use of which is advancing exponentially both among professionals and lay people, produced unacceptable high rates of erroneous information on infectious diseases in special populations [27].

In conclusion, a group of committed health professionals providing joint efforts under the umbrella of the One Health approach might contribute with high-level scientific collaborations to improve the decisions of the European stakeholders and to provide assistance for frontline healthcare workers involved in the infectious disease-related challenges of the vulnerable populations.
